# Introduction to Editorial Board Members: Professor David A. Tirrell

**DOI:** 10.1002/btm2.10040

**Published:** 2016-10-31

**Authors:** Julie Champion

**Affiliations:** ^1^ Associate Professor, Tanner Faculty Fellow, School of Chemical & Biomolecular Engineering Georgia Institute of Technology Atlanta GA 30332

In this second issue of *Bioengineering and Translational Medicine*, we are pleased to introduce our Editorial Board Member, Prof. David A. Tirrell. Prof. Tirrell is the Ross McCollum‐William H. Corcoran Professor of Chemistry and Chemical Engineering and Director of the Beckman Institute at California Institute of Technology. He earned his BS degree in Chemistry at Massachusetts Institute of Technology and his PhD in Polymer Science and Engineering at University of Massachusetts Amherst.

Professor Tirrell's research has made a deep impact on multiple fields including synthetic polymers, membranes, and artificial proteins. His pioneering research in artificial proteins has been particularly inspiring. Artificial proteins are hybrids between synthetic polymers and natural proteins. They may contain amino acids beyond the canonical 20 in the genetic code, and/or amino acid sequences not found in naturally occurring proteins, which endow these proteins with functions well beyond their natural counterparts. Since artificial proteins are produced from artificial genes by cellular transcription and translation processes, they have defined length, sequence, and structure that enable functions such as binding and catalysis, which are not possible by inherently heterogeneous synthetic polymers. Professor Tirrell has leveraged these advantages to produce a wide variety of new macromolecules with novel structure and function by developing methods to incorporate non‐natural amino acids with different chemical functionalities and careful design of amino acid sequences that combine multiple functional domains.^1^

Professor David Tirrell (front row, center) pictured with many of his current and former students and postdocs at the symposium held in honor of his 60th birthday.

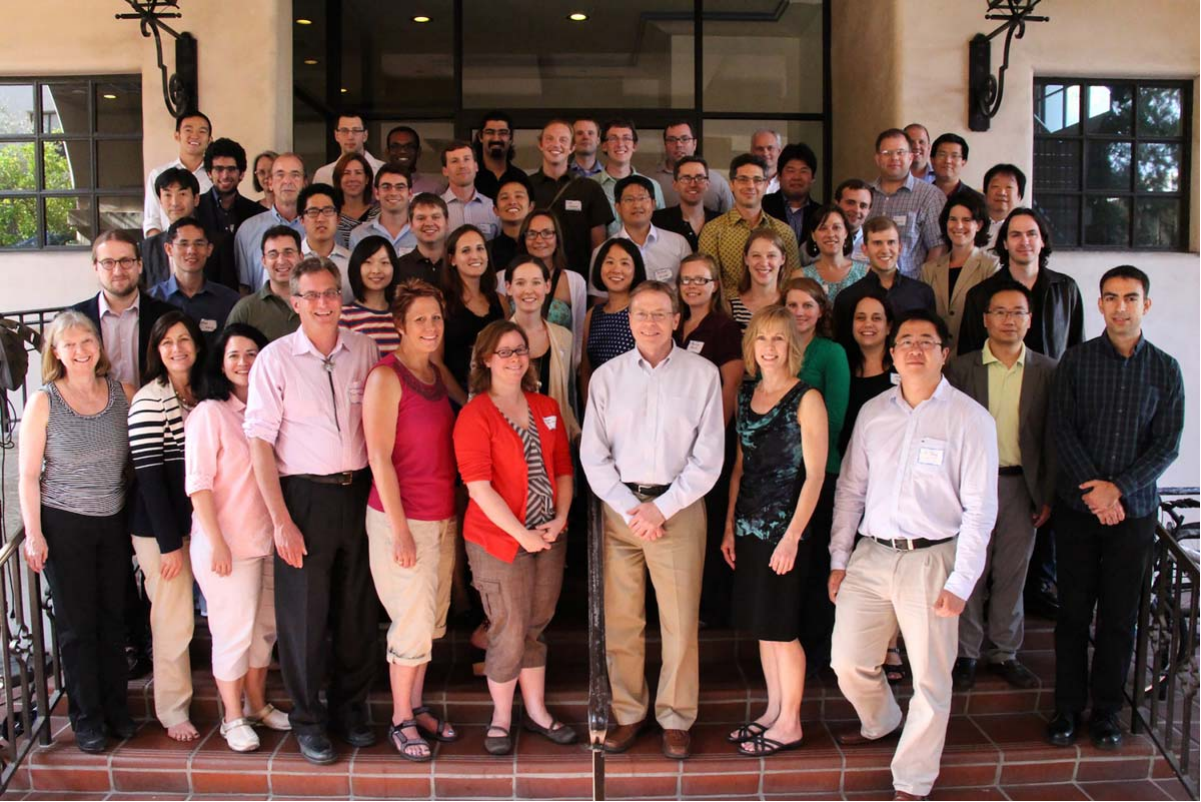

Artificial proteins produced in the Tirrell Lab have made significant contributions to the field of biomaterials.^2^ The wide diversity of functions incorporated into the proteins is reflected in the wide range of tunable properties of the materials prepared from these proteins. Protein hydrogels, for example, can be cross‐linked by covalent bonds between amino acids, by noncovalent affinity‐interactions between coiled‐coil protein domains, or a combination.^3^ These materials can be used to support cells for tissue engineering applications through incorporation of cell binding domains, protease cleavage sites, and other factors. Professor Tirrell's protein materials have made significant impact beyond biomedicine, in particular in the areas of soft matter and cellular biophysics by providing new insights beyond those possible with traditional methods and materials.^4^, ^5^

Professor Tirrell has also advanced artificial proteins as a powerful tool for proteomics. He developed a technique referred to as bioorthogonal noncanonical amino acid tagging (BONCAT).^6^ By engineering various components of the protein expression machinery, cells can incorporate non‐natural amino acids into their proteins during expression which can then be detected by methods such as mass spectrometry or fluorescence microscopy. BONCAT can be applied selectively, for example, so that incorporation only occurs in response to a natural stimulus, or only occurs in one cell type within a mixture of cells or even a living organism. The impact of Professor Tirrell's research is evident from the wide range of applications that BONCAT and associated techniques have enabled. In one example, protein expression patterns in the neurons of live *C. elegans* were studied, while in another a new protein was identified that is important in the virulence of a bacterial pathogen, *P. aeruginosa*.^7^, ^8^

Professor Tirrell's contributions to macromolecular chemistry and engineering have been recognized by numerous awards and honors including his election to the National Academy of Sciences, the National Academy of Engineering, the National Academy of Medicine, and the American Academy of Arts and Sciences. He has been recognized as the Arthur C. Cope Scholar and has received Carl Marvel award, Harrison Howe award, S. C. Lind and Madison Marshall Award of the American Chemical Society, as well as the ACS Award in Polymer Chemistry. He holds the Chancellor's Medal of the University of Massachusetts, the G. N. Lewis Medal of the University of California Berkeley, and the degree of Doctor honoris causa from the Technical University of Eindhoven.

These honors reflect the deep scientific impact of Prof. Tirrell's research, yet his impact through mentoring the next generation of scientists and engineers is just as impressive. Over the years Prof. Tirrell has trained a large number of students and post‐docs that are further advancing the scientific frontier in macromolecular chemistry, biomaterials, and protein design. On behalf of all his former and current students, I express my admiration for his contributions to science and sincere appreciation for his commitment to nurturing scientists throughout their careers.

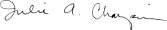


Julie Champion
Associate Professor
Tanner Faculty Fellow
School of Chemical & Biomolecular Engineering
Georgia Institute of Technology
Atlanta, GA 30332
Email: julie.champion@chbe.gatech.edu
